# The Immunomodulary Effects of Systematic Exercise in Older Adults and People with Parkinson’s Disease

**DOI:** 10.3390/jcm9010184

**Published:** 2020-01-09

**Authors:** Jadwiga Szymura, Jadwiga Kubica, Magdalena Wiecek, Joanna Pera

**Affiliations:** 1 Department of Clinical Rehabilitation, Faculty of Motor Rehabilitation, University of Physical Education in Krakow, 31–571 Krakow, Poland; 2 Institute of Physiotherapy, Faculty of Health Science, Jagiellonian University Medical College, 31–126 Krakow, Poland; 3 Department of Physiology and Biochemistry, Faculty of Physical Education and Sport, University of Physical Education in Krakow, 31–571 Krakow, Poland; magdalena.wiecek@awf.krakow.pl; 4 Department of Neurology, Faculty of Medicine, Jagiellonian University Medical College, 31–503 Krakow, Poland; joanna.pera@uj.edu.pl

**Keywords:** inflammation, regular training, neurological disorders, older people

## Abstract

We sought to investigate whether regular balance training of moderate intensity (BT) has an effect on changes in selected cytokines, neurotrophic factors, CD200 and fractalkine in healthy older adults and participants with Parkinson’s disease (PD). Sixty-two subjects were divided into groups depending on experimental intervention: (1) group of people with PD participating in BT (PDBT), (2) group of healthy older people participating in BT (HBT), (3,4) control groups including healthy individuals (HNT) and people with PD (PDNT). Blood samples were collected twice: before and after 12 weeks of balance exercise (PDBT, HBT), or 12 weeks apart (PDNT, HNT). The study revealed significant increase of interleukin10 (PDBT, *p* = 0.026; HBT, *p* = 0.011), β-nerve growth factor (HBT, *p* = 0.002; PDBT, *p* = 0.016), transforming growth factor-β1 (PDBT, *p* = 0.018; HBT, *p* < 0.004), brain-derived neurotrophic factor (PDBT, *p* = 0.011; HBT, *p* < 0.001) and fractalkine (PDBT, *p* = 0.045; HBT, *p* < 0.003) concentration only in training groups. In PDBT, we have found a significant decrease of tumor necrosis factor alpha. No training effect on concentration of interleukin6, insulin-like growth factor 1 and CD200 was observed in both training and control groups. Regular training can modulate level of inflammatory markers and induce neuroprotective mechanism to reduce the inflammatory response.

## 1. Introduction

Physical health in older adults is compromised by age-related changes in health status and functionality. Human aging is associated with a progressive decline in functioning of the immune system, resulting in low-grade inflammation called inflamm-aging. A major characteristic of this process is chronic activation of the innate immunity and increased levels of circulating pro-inflammatory cytokines [1,2].

Systemic inflammation connects with the brain via bidirectional neural or humoral pathways [3]. It has been suggested in studies that the interleukin 1 beta (IL-1β) is a key link in this communication. Circulating cytokines and inflammatory mediators may contact with macrophages in the regions of the central nervous system (CNS) lacking a typical tight blood–brain barrier [4]. The immune–brain route of communication involves signalling from mediators in the blood to the cerebral endothelial cells, next to the perivascular macrophages which, in turn, provide a signal for the microglia.

Microglia are involved in the immune response against viruses and unicellular organisms that may cause CNS damage. Infection, ischemia and trauma may mediate an acute neuroinflammatory response and microglial activation. Secretion of neurotrophic factors such as brain-derived neurotrophic factor (BDNF), glial cell-derived neurotrophic factor (GDNF), nerve growth factor (NGF), insulin-like growth factor 1 (IGF-1) or the vascular endothelial growth factor (VEGF) by microglia is neuroprotective. In the healthy brain, microglia exist in a resting state, but when exposed to pathological insult, microglia transform from resting to a spectrum of activated stages [5]. The classical activation state (M1) is associated with a pro-inflammatory phenotype having enhanced production of the tumour necrosis factor (TNF), interleukins 6 (IL-6), IL-12, IL-1β and several chemokines such as chemokine (C-C motif) ligand 2 (CCL2) and the human interferon-inducible protein 10 (IP-10 or CXCL10). Alternatively activated microglia (M2) are associated with healing and scavenging. This phenotype is related to the increased expression of trophic factors and anti-inflammatory cytokines such as interleukin-10 (IL-10), the transforming growth factor β (TGF-β), IGF, NGF-β or BDNF [6,7].

The activity of microglia can be inhibited by anti-inflammatory molecules [5]. One of them is fractalkine, which has been reported to inhibit the release of inflammatory cytokines while reducing microglia activation [8]. Fractalkine can be either neuroprotective or neurotoxic depending on the M1 or M2 activation state [9]. M1 microglia exacerbate inflammation and mediate neurodegeneration. A number of neurodegenerative diseases are associated with a chronic neuroinflammatory process. Moreover, there is a growing body of evidence suggesting that peripheral inflammation can activate microglia, which, in turn, can induce or trigger stronger responses that aggravate neurodegenerative processes and may contribute to the aetiology and progression of Parkinson’s disease (PD) [10]. It has been reported that in the serum and cerebrospinal fluid of PD patients, the increase in M1 associated with cytokines such as TNF and IL-6 is possibly induced in response to the TNF activation of astrocytes [11].

Physical exercise is a potential strategy to counteract immune system function and chronic inflammation that accompanies aging. Exercise improves neuroplasticity by modulating multiple systems involved in the regulation of neuroinflammation. Regular exercise is associated with an increase in synaptic and cerebrovascular plasticity [12], as well as improved anti-oxidative capacity [13,14]. During exercise, local immune adaptations occur in the skeletal muscles [15], inducing a release of IL-6 [16]. This cytokine is produced in response to muscle contraction and acts both as pro-inflammatory and anti-inflammatory [17]. The increase in IL-6 concentration is exponential but proportional to the duration and the amount of muscle mass involved in the exercise, which is suggested to induce the anti-inflammatory cytokine cascade [14,18].

Regular exercise modulates metabolic changes in the peripheral immune system by downregulation of circulating inflammatory cytokines, for example, TNFα, and IL-1β, and upregulation of anti-inflammatory cytokines such as interleukin IL-10 [19]. Resistance training has minimal effects on resting inflammatory or acquired immune parameters, as assessed by analysis of peripheral blood [1,20,21]. In turn, moderate physical exercise in older adults promotes the modulation of inflammation by reducing TNF-α and IL-6 levels, while increasing IL-10 levels [22]. More significant changes follow aerobic exercise training [1,23].

Moreover, physical activity influences neurotrophic factor blood expression both in healthy persons and in patients with PD [24,25]. The neuroprotective role of NGF, BDNF and IGF-1 in response to exercise has been explored in aging and neurodegenerative diseases. Upregulation of NGF has mainly been studied in animal models with Alzheimer’s disease [26]. In human studies, lack of changes in NGF levels was noted among older women in response to resistance training intervention, while some upregulation was detected in patients with multiple sclerosis (MS).

Neurotrophic factors are important indicators of enhanced neuroplastic activity both in healthy individuals and in patients with the neurological disorders, thus, further studies on the possible exercise effects are warranted.

With aging, changes of postural stability, which are indicated by an increased postural sway, inability to execute effective stepping responses, higher risk of falls, and related to injuries morbidity, are observed [27]. Balance impairment and postural instability are also the common incapacitating symptoms of PD, which usually occur in the middle–later stages of the disease [28]. Results from systematic reviews indicate that balance training may have a positive effect on treating postural instability among people with PD and healthy older adults [28,29,30].

The aim of the authors’ research was to determine whether regular balance training of moderate intensity has an effect on changes in selected cytokines, neurotrophic factors and proteins such as CD200 and fractalkine in healthy older adults and participants with PD.

The authors hypothesized that the conducted 12 week balance training has a positive effect on the concentration of anti-inflammatory cytokines, neurotrophic factors, CD200 and fractalkine, and at the same time, it induces a reduction in pro-inflammatory cytokines for both training groups.

## 2. Materials and Methods

### 2.1. Study Participants

The study involved older healthy people and individuals with idiopathic PD. The inclusion criteria for subjects with PD were age ≥60 years, diagnosis of idiopathic PD (Hoehn and Yahr stage between 2 and 3), no changes regarding the applied pharmacotherapy in the month preceding the test, no orthopaedic conditions limiting physical exercise or deep brain stimulation surgery, independent gait, and physical fitness enabling participation in the training programme. Inclusion criteria for healthy older people were age ≥60 years, no medication affecting the functioning of CNS (e.g., neuroleptics, antidepressants), no neurological or orthopaedic disorders limiting physical exercise, independent gait and physical fitness allowing participation in the training programme.

Exclusion criteria were lack of informed consent to participate in the study, musculoskeletal injuries (e.g., fractures and prostheses), diabetes, diagnosed dementia, (Mini Mental State Examination <25), previous stroke or severe traumatic brain injury, other CNS diseases and participation in regular physical exercises.

Sixty-two subjects were randomly divided into groups depending on experimental intervention: (1) group of people with PD participating in moderate-intensity exercise (PDBT), (2) group of healthy older people participating in moderate-intensity exercise (HBT), (3) control group including individuals with PD (PDNT) and (4) control group comprising healthy people (HNT). After completion of the study, each of the control groups could take part in the 12 week balance training programme.

### 2.2. Assessment Procedure

All of the participants underwent neurological examination before inclusion in the study. Initial assessment also involved an interview and basic anthropometric measurements (such as gender, age, body mass and height, and physical activity). Venous blood samples were collected twice: before (T1) and after 12 weeks of balance exercise (T2) (PDBT and HBT groups), or 12 weeks apart (T2) (PDNT and HNT groups). T1 was carried out 2 days before the beginning of the exercise programme. At the same time, pre-study data (T1) were also collected in the control group. T2 was performed 2 days after the completion of the 12 week balance training programme (PDBT and HBT groups) or after 12 weeks (groups PDNT, HNT). T1 and T2 were performed at the same time of the day for each participant. All participants with Parkinson’s disease were evaluated during the ON phase (good response to medication).

### 2.3. Ethical Approval

The study was conducted with the permission of the Local Ethical Committee (OIL/KBL/7/2019), according to the principles established in the Declaration of Helsinki for research with human subjects’ participation. The volunteers were informed about the procedures and purpose of the research in detail, and about the possibility of resigning from participation at any stage without giving reasons. All volunteers provided written consent for participation in the study, as well as for the use of personal data and research results for scientific purposes.

### 2.4. Anthropometric Measurements and Body Composition Assessment

Anthropometric measurements were performed in the morning, approximately 20 min before blood sampling. Body mass (Jawon Medical IOI-353, Gyeongsan, Korea) and body height (Seca 217, Hamburg, Germany) for each participant were measured using standard procedures.

### 2.5. Balance Training with Moderate-Intensity Exercise

Participants from training groups took part in the 12 weeks training programme under the supervision of a physiotherapist. There were three therapy sessions per week (every other day; 36 sessions in sum). In order to gradually adapt participants to the training, in the first week, training sessions lasted 30 min and were then extended to 60 min. The session was divided into three stages: (1) 5 min warm up, (2) 50 min of balance training (in the first week, 20 min), (3) 5 min cool down. The specification of therapy session was previously explained by Kubica et al. [31].

#### Heart Rate and Estimation of Maximal Heart Rate

In this study, heart rate was monitored using a heart rate monitor (Polar RS300, Polar Electro Oy, Kempele, Finland). Maximal heart rate (HR_max_) was predicted for each participant using the following formula: HR_max_ = 208 − 0.7 × age, developed by Tanaka et al. [32].

### 2.6. Postural Control Measurement

To assess postural control, the Tinetti Performance-Oriented Mobility Assessment (POMA) was used [33]. The individual score of each person is the combination of three measures: the overall gait assessment score (POMA-G, 12 points), the overall balance assessment score (POMA-B, 16 points) and the gait and balance score (i.e., Tinetti total score of 28 points, POMA).

### 2.7. Blood Sampling

Venous blood samples were collected by venipuncture from the antecubital vein at rest in the morning hours (8–10 a.m.) using the BD Vacutainer^®^ vacuum system (Becton Dickinson, Franklin Lakes, NJ, USA).

Blood for biochemical parameters analysed in the plasma (i.e., IL-6, IL-10, and TNFα) was collected into tubes containing ethylenediaminetetraacetic acid (EDTA), and was then centrifuged (relative centrifugal force 1.000× *g*) directly after collection for 15 min at 4 °C using the MPW-351R centrifuge (MPW Med. Instruments, Warsaw, Poland).

Blood for biochemical parameters analysed in the serum (i.e., BDNF, TGF-β1, IGF-1, β-NGF, CD200 and fractalkine concentrations) was collected into test tubes with a clotting activator and was left to clot for a minimum of 30 min at 20–22 °C, and was then centrifuged (relative centrifugal force 1.000× *g*) for 15 min at 4 °C using the MPW-351R centrifuge (MPW Med. Instruments, Warsaw, Poland).

The obtained plasma and serum were stored at −75 °C until analysis (ULUF 450 Arctiko, Esbjerg, DK).

### 2.8. Measurements of Biochemical Parameters

Determination for all biochemical parameters was performed using the immunoenzymatic method (ELISA) with absorbance measurement performed via the E-LizaMat 3000 microplate reader (DRG International, Inc., Springfield, NJ, USA) according to the methodology presented by the manufacturer, reading the results from the standard curve created during each test (R&D Systems, Minneapolis, MN, USA, or Thermo Fischer Scientific, Waltham, MA, USA).

The plasma concentrations of IL-6 were determined using the HS600C Human ELISA high-sensitivity test (R&D Systems Inc., Minneapolis, MN, USA). The detection limit was 0.031 pg/mL. The intra- and inter-assay coefficients of variation for this measurement were <4.7% and 10.8%, respectively.

The plasma concentrations for IL-10 were determined using the Human ELISA high-sensitivity test HS100C (R&D Systems Inc., Minneapolis, MN, USA). The detection limit was 0.09 pg/mL. The intra- and inter-assay coefficients of variation for this measurement were <9.4% and 12.8%, respectively.

The plasma concentrations for TNF-α were determined using the HSTA00E Human ELISA high-sensitivity test (R&D Systems Inc., Minneapolis, MN, USA). The detection limit was 0.022 pg/mL. The intra- and inter-assay coefficients of variation for this measurement were <2.2% and 6.7%, respectively.

The serum concentration of BDNF was determined using the DBNT00 Human ELISA test (R&D Systems Inc., Minneapolis, MN, USA). The detection limit was 0.997 pg/mL. The intra- and inter-assay coefficients of variation for this measurement were <3.2% and 7.2%, respectively.

The serum concentration of β-NGF was determined using the EHNGF Human ELISA test (Thermo Fischer Scientific, Waltham, MA, USA). The detection limit was 14 pg/mL. The intra- and inter-assay coefficients of variation for this measurement were <10% and 12%, respectively.

The serum concentration of TGF-β1 was determined using the DB100B Human ELISA test (R&D Systems Inc., Minneapolis, MN, USA). The detection limit was 4.61 pg/mL. The intra- and inter-assay coefficients of variation for this measurement were <2.9% and 9.1%, respectively.

The serum concentration of IGF-1 was determined using the DB100B Human ELISA test (R&D Systems Inc., Minneapolis, MN, USA). The detection limit was 0.026 ng/mL. The intra- and inter-assay coefficients of variation for this measurement were <4.3% and 8.3%, respectively.

The serum concentration of CD200 was determined using the EHCD200 Human ELISA test (Thermo Fischer Scientific, Waltham, MA, USA). The detection limit was 20 pg/mL. The intra- and inter-assay coefficients of variation for this measurement were <10% and 12%, respectively.

The serum concentration of fractalkine was determined using the DCX310 Human ELISA test (R&D Systems Inc., Minneapolis, MN, USA). The detection limit was 0.018 ng/mL. The intra- and inter-assay coefficients of variation for this measurement were <3.2% and 8.9%, respectively.

### 2.9. Statistical Analysis

Normal distribution of variables was evaluated using the Shapiro–Wilk test. Homogeneity of variance was tested using the Levene test. Data are reported as mean ± standard deviation (SD) or median and interquartile range (IQR) where applicable. Statistical analysis was conducted using STATISTICA (version 13.0 for Windows, StatSoft, Inc., Tulsa, OK, USA). The significance of differences between groups in the case of individual measurements was assessed using tests for independent samples (ANOVA F analysis of variance test or ANOVA, the Kruskal–Wallis nonparametric test, the Student’s *t*-test or the Mann–Whitney U nonparametric test). Analysis of variance with repeated measurements was used to compare the influence of balance training on changes in the analysed variables. In the case of a significant influence of any of the main factors (i.e., GROUP, BT (balance training) or GROUP × BT interactions), the significance of differences between specific averages was checked by performing statistical analysis for planned comparisons: Fisher’s exact test based on the Student’s *t*-test (post-hoc). In order to quantify the dependence of selected cytokines, neurotrophic factors, CD200 and fractalkine on postural control in the studied groups, Spearman’s rank correlation coefficient was used. The *p* value of <0.05 was considered statistically significant.

## 3. Results

### 3.1. Characteristics of the Participants

The study comprised 61 participants. Their baseline characteristics are summarised in Table 1. Subjects who had ≥3 absences from training sessions (*n* = 0) and PD patients with pharmacotherapy modified during the study were excluded (*n* = 1). For the 29 participants with PD analysed in this study, the mean age was 65.69 ± 7.48 years, and 10 (35%) were women. However, among the 32 healthy (H) participants, the mean age was 66.47 ± 3.21 years, and 12 (38%) were women. No significant differences in the main characteristics such as age, gender, height, mass or BMI were found between participants with PD or the remaining participants enrolled in the trial.

### 3.2. Baseline Characteristics of Postural Control

The baseline POMA results were significantly lower (*p* < 0.001) in all PD participants compared with the whole healthy participants (Table 2).

### 3.3. Training Effects on Postural Control

Analysing the POMA (Tinetti total score) results, significant group-related differences (ANOVA, GROUP factor) were found (F = 58.05, *p* < 0.01), as well as a significant BT effect regarding changes in POMA results (F = 8.22, *p* = 0.01), while the changes in POMA following BT differed (F = 12.72, *p* = 0.01) (GROUP × BT). In post-hoc analysis, it was shown that the POMA results before BT were higher in the healthy elder individuals (respectively: HBT, and HNT groups) compared with persons with PD (respectively, PDBT group: *p* < 0.001, *p* < 0.001, and the PDNT group: *p* < 0.001, and *p* < 0.001). After BT, the POMA results were still higher in the HBT and HNT groups compared with the PDBT group (respectively, *p* < 0.001 and *p* < 0.001), as well as the PDNT group (respectively, *p* < 0.001 and *p* < 0.001). The POMA results after BT were significantly lower in the PDNT group compared with the PDBT group (*p* < 0.001). Twelve weeks of BT with moderate intensity exercises induced significant changes in the POMA results in the PDBT (*p* < 0.001) and HBT groups (*p* < 0.001). POMA results for the PDNT and the HNT groups were similar at baseline and after 12 weeks of training (Table 2).

### 3.4. Baseline Characteristics of Biochemical Indicators

The baseline IL-6, IL-10, TNF-α, BDNF, TGF-β1, CD200 and fractalkine concentrations were comparable in the All PD group and in the All H group (*p* > 0.05).

The baseline concentration of β-NGF was significantly lower (*p* < 0.001) in the PD compared with the All H group, while IGF-I was significantly higher (*p* = 0.032) in the All PD group compared with the All H group (Table 3).

### 3.5. Training Effects on Biochemical Indicators

The numerical values of the analysed variables and the results of statistical analysis in the PDBT, PDNT, HBT and HNT groups, as well as for baseline values in the All PD and All H groups are shown in Table 3.

#### 3.5.1. Training Effects on Plasma Interleukin-6 Concentration

The IL-6 concentration did not differ significantly (ANOVA) before or after completion of the 12 week BT with moderate intensity exercise or between groups (*p* < 0.05).

#### 3.5.2. Training Effects on Plasma Interleukin-10 Concentration

**Figure 1:**
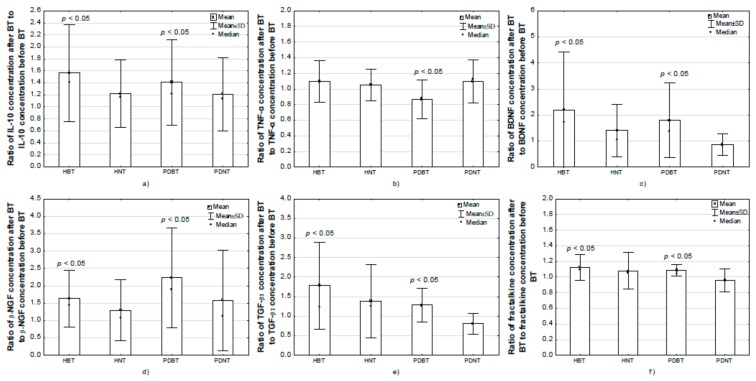
Comparison of changes in selected cytokines and neurotrophic factors as well as CD200 proteins and fractalkine concentration after moderate-exercise balance training in the studied groups: (**a**) change in plasma IL-10; (**b**) change in plasma TNF-α; (**c**) change in serum BDNF; (**d**) change in serum β-NGF; (**e**) change in serum TGF-β1; (**f**) change in serum fractalkine. Data presented as mean ± SD and median; SD: standard deviation; IL-10: interleukin 10; TNF-α: tumor necrosis factor alpha; BDNF: brain-derived neurotrophic factor; β-NGF: beta nerve growth factor; TGF-β1: beta 1 transforming growth factor.

Significant group-related differences (ANOVA, BT factor) in IL-10 concentration were found (F = 10.05, *p* < 0.01). Differences between groups regarding changes in IL-10 concentration after BT with moderate-intensity exercise (GROUP × BT) were found (F = 2.41, *p* = 0.07). In post-hoc analysis, it was shown that the concentration of IL-10 before BT was similar in all of the compared groups. The concentration of IL-10 after BT was higher in the HBT compared with the HNT (*p* = 0.025) and PDNT groups (*p* = 0.014). After BT, the concentration of IL-10 increased compared with the baseline value in the PDBT (*p* = 0.026) and HBT (*p* = 0.011) groups (Figure 1).

#### 3.5.3. Training Effects on Plasma TNF-α Concentration

Significant group-related differences (ANOVA, GROUP factor) in TNF-α concentration were found (F = 4.09, *p* = 0.01). Differences between groups were also found regarding changes in TNF-α concentration after BT with moderate intensity (GROUP × BT) (F = 2.84, *p* = 0.07). In post-hoc analysis, it was demonstrated that the concentration of TNF-α after BT (*p* = 0.046) was higher in the HNT compared with the HBT (*p* = 0.024), PDBT (*p* = 0.008) and PDNT (*p* = 0.003) groups. After completion of the 12 week BT, the concentration of TNF-α decreased compared with the baseline value in the PDBT group (*p* = 0.001) (Figure 1).

#### 3.5.4. Training Effects on Serum BDNF Concentration

In the compared groups, significant effects of BT with moderate intensity exercise were found in the case of BDNF concentration changes (F = 11.52, *p* < 0.01) and significant differences between groups were noted regarding changes in BDNF concentration after BT (GROUP × BT) (F = 4.54, *p* = 0.01) (ANOVA). In post-hoc analysis, it was shown that after BT, the concentration of BDNF increased compared with the baseline values in the PDBT (*p* = 0.011) and HBT (*p* < 0.001) groups (Figure 1). In the nontraining groups, no changes in BDNF concentration were observed (*p* > 0.05).

#### 3.5.5. Training Effects on Serum β-NGF Concentration

In the analysed groups, significant differences between groups (GROUP factor) were noted in β-NGF concentration (F = 12.92, *p* < 0.01), as well as a significant effect of BT with moderate intensity exercise regarding β-NGF concentration changes (F = 6.06, *p* = 0.02) (ANOVA). In post-hoc analysis, it was demonstrated that β-NGF concentration before BT was significantly higher in the HBT and HNT groups compared with PDBT (respectively, *p* = 0.001 and *p* < 0.001) and PDNT (respectively, *p* = 0.005and *p* = 0.001). Similar relationships in the analysed groups were noted after BT. β-NGF concentration after BT was significantly higher in the HBT and HNT groups compared with PDBT (respectively, *p* < 0.001 and *p* = 0.007) and PDNT (respectively, *p* < 0.001 and *p* < 0.001). After BT, the concentration of β-NGF increased compared with the baseline value in the HBT (*p* = 0.002). After BT, the β-NGF concentration significantly increased in the PDBT group (*p* = 0.016) (Figure 1), however, its value was still lower than in the HBT group.

#### 3.5.6. Training Effects on Serum TGF-β1 Concentration

In the analysed groups, significant differences were found between groups regarding changes in TGF-β1 concentration after BT with moderate intensity exercise (GROUP × BT) (F = 4.65, *p* < 0.01) (ANOVA). In post-hoc analysis, it was shown that after BT, the concentration of TGF-β1 increased compared with the baseline value in the PDBT (*p* = 0.018) and HBT (*p* < 0.004) groups (Figure 1). In the nontraining groups, no changes in TGF-β1 concentration were observed (*p* > 0.05).

#### 3.5.7. Training Effects on Serum IGF-1 Concentration

Analysing the results in the compared groups (ANOVA), no significant differences between them were found regarding IGF-I concentration. In none of the groups did the BT with moderate intensity exercise have significant impact on IGF-I concentration changes (*p* > 0.05).

#### 3.5.8. Training Effects on Serum CD200 Concentration

CD200 concentration did not differ significantly before the 12 week BT with moderate intensity exercise or between groups (*p* < 0.05). Balance training did not affect the change in CD200 concentration among the groups under study (*p* > 0.05).

#### 3.5.9. Training Effects on Serum Fractalkine Concentration

In the compared groups, significant effects of BT with moderate intensity exercise (ANOVA) were noted regarding fractalkine concentration changes (F = 5.20, *p* = 0.02) as well as significant differences between groups concerning changes in fractalkine concentration after BT (GROUP × BT) (F = 3.04, *p* = 0.03). In post-hoc analysis, it was shown that after BT, the concentration of fractalkine increased compared with the baseline value in the PDBT (*p* = 0.045) and HBT (*p* = 0.003) groups (Figure 1). In the nontraining groups, no changes in fractalkine concentration were observed (*p* > 0.05).

### 3.6. Correlations

There was a significant (*p* < 0.05) negative correlation between the baseline POMA results and the baseline IL-6 concentration (r = −0.45) in subjects with PD, as well as a significant (*p* < 0.05) negative correlation between the baseline POMA results and the baseline BDNF concentration (r = −0.46) in healthy individuals. Additionally, a significant (*p* < 0.05) positive correlation was found between changes in POMA score and IL-10 concentration as a result of 12 weeks balance training (r = 0.47) in all training participants.

## 4. Discussion

In the present study, we investigated the effects of 12 weeks balance training of moderate intensity on selected cytokines, neurotrophic factors, CD200 and fractalkine in healthy older adults and people with PD.

The main findings from this study are that twelve weeks of BT with moderate intensity exercises improved the POMA results in the training groups, although the HBT group results were still higher compared with those of the PDBT group. The results revealed a positive correlation between changes in POMA score and IL-10 concentration in both training groups.

Further, we found that systematic balance training of moderate intensity influenced anti- and pro-inflammatory cytokines and neurotrophic factors levels. After completion of the 12 weeks training programme, the concentrations of IL-10, β-NGF, TGF-β1, BDNF and fractalkine increased in both training groups, but not in the nontraining groups. Only in the PDBT was a significant decrease found in TNF-α. No training effects on concentration of IL-6, IGF-1 or CD200 were observed in both the training and control groups (Table 3, Figure 1).

Regular exercise is important for maintaining general health status [34]. Physical activity is meant to be a factor that accelerates the increase of bodily functions [9] with a positive impact on balance control [30]. Apart from improving neuroplasticity, physical activity can also modulate multiple systems that are known to regulate neuroinflammation and glial activation [5]. It has been shown that exercise training induces transient changes in immunity responses. Exercises are considered as crucial modulators that may regulate the immune system and prevent premature immunosenescence. Systematic activity is a safe mode of intervention in preventing chronic low-grade inflammation in older people [35]. It is still debated whether the magnitude of changes in immune cell levels is dependent on exercise intensity. It has been revealed in studies that regular exercises of moderate to vigorous intensity lasting less than 60 min are important to enhance immune defence activity and metabolic health [36,37,38], nonetheless, heavy bouts of activity may even have the opposite effect [39].

Long-lasting aerobic activity and resistance exercise have been shown to induce an increase of circulating factors such as IGF-1, BDNF and NGF, which may affect brain plasticity in physiological aging or neurodegenerative pathologies. In neurological disorders, exercise training can provide participants with specific clinical benefits, but only if repeated habitually over a period of time [40].

NGF production is increased in inflammatory diseases and induced by a variety of pro- and anti-inflammatory cytokines such as IL-1, IL-4, IL-5, TNF-α, TGF-β and interferon-beta (IFN-β). NGF and other trophic factors in CNS play a role in providing protection against inflammation-related neuronal damage caused by pro-inflammatory cytokines [41].

Peripheral sources of IGF-I supplied to the brain can mediate the effect of exercise on neuronal plasticity. The IGF-I pathway is important for nerve growth and differentiation. The role played by IGF-I during exercise may be associated with the action of BDNF, as IGF-I entrains similar downstream pathways to BDNF action [42]. Together, they are considered as key factors concerning the effects of exercise on learning and memory. The release of BDNF and IGF-1 in response to training has been studied before, however, the evidence was inconsistent. In the authors’ study, confirmation has not been found for the proposed link between these growth factors. A possible reason explaining the IGF-1 results is the influence of body health factors directly related to exercise, such as nutrition and glucose metabolism, which were not measured in the study. Furthermore, as showed by Berg and Bang [43], increases in blood IGF-1 have been found to occur only during the training period. The level of IGF-1 drops immediately within 10 min after training, suggesting that the effect is only transient.

The exercise-induced increase of BDNF depends on the intensity and duration of exercises [34,44]. Moderate intensity training seems to be the most effective for inducing changes in BDNF concentration, also in participants with PD [44,45]. In the research by Zoladz et al. [44], it was revealed that the applied training programme resulted in an increase of serum BDNF and a decrease of plasma TNF-α levels, indicating that training induced some anti-inflammatory responses in PD patients, although a control group was not included. We have observed similar results in this study, in which systematic balance training of moderate intensity had a positive effect on the BNDF level in both training groups, while inducing a decrease of TNF-α concentration in the PDBT group. No significant changes in BDNF concentration were found in the groups without training interventions. These results are in line with previous studies, in which sedentary, inactive subjects showed lower or even no response in BNDF level following one session comprising acute exercises of different intensities, while the greatest response was reported in well-trained individuals [46]. It was postulated that the expression of specific BDNF transcripts may be affected by immune and inflammation processes [47], as BNDF is considered to have an indirect neuroprotective effect on microglial activation by reduction of neuronal injury and inflammation.

Deregulation of immunity observed with age is related to increased susceptibility to infections, autoimmune and metabolic diseases as well as neurologic disorders [38]. One of the biological events characterising this phenomenon called immunosenescence includes alteration in inflammatory and oxidation state with an increase of pro-inflammatory cytokines such as IL-1, IL-6, TNF-α and the C reactive protein (CRP) [48]. Aging and systemic inflammation are considered as the main risk factors for several neurodegenerative diseases. Activated microglia and pro-inflammatory cytokines play active roles in the pathogenesis of neurodegenerative diseases such as Alzheimer’s disease (AD) or PD [5,49]. Degeneration of dopaminergic neurons can activate microglia, leading to the release of different inflammatory factors, such as pro-inflammatory cytokines or chemokines (TNF-α, IL-1β, IL- 6 and interferon-γ (IFN-γ)) [50]. Thus, the way to reduce cell loss in PD may be by changes in inflammatory state.

Microglia activation can be regulated by TGF-β1, an anti-inflammatory cytokine essential for microglia homeostasis [51]. TGF-β1 is considered an important endogenous factor, inhibiting M1 activation and shifting microglia phenotypes towards M2 activation. Moreover, TGF-β1 is neuroprotective in brain injury and inflammation, inducing tissue repair [52]. In studies by Chen et al. [53] it has been shown that the activity of microglia in vitro is modulated by TGF-β1, decreasing TNF-α, IL-1β, NO or rective oxygen speciesproduction induced by 1-methyl-4-phenylpyridinium (MPP+) dopaminergic neuronal loss in the rat model of PD. The authors have demonstrated a significant decrease in TNF-α and an increase of TGF-β1 in response to regular balance exercises in the PDBT group, indicating that the conducted training had a modulatory effect on the inflammation process in participants with PD. After completion, the training participants from the HBT group had significantly lower levels of TNF-α compared with the HNT group, and higher levels of TGF- β1 in comparison with baseline, showing that regular balance training of moderate intensity, through neuroprotection of TGF- β1, can positively affect pro-inflammation induced by TNF-α.

Il-6 influences microglia, mediating neuroprotective or neurotoxic responses. In some studies, elevated levels of IL-6 during CNS disorders have been noted, while in others, it has been demonstrated that IL-6 has neuroprotective properties [16]. The release of IL-6 can be regulated by physical exercise, since muscle contraction, mediated by exercise training, produces and releases myokines [54]. Increased production of pro-inflammatory cytokines such as IL-1β, TNF-α and IL-6 was observed 24 h after eccentric training, leading to a local pro-inflammatory response. That inflammatory response was explained as necessary in the process of healing and tissue debris [55]. In literature on the subject, the increase of IL-6 is suggested to precede the expression of anti-inflammatory cytokines (IL-1ra and IL-10), indicating that IL-6 induces an anti-inflammatory cytokine cascade [56]. The results obtained by the authors of this study did not confirm that. Although the concentration of IL-10 significantly increased after completion of balance training in both training groups, the conducted training did not significantly alter the level of IL-6. A possible explanation for the lack of changes in IL-6 concentration might be the kind of exercises proposed in the study—mainly concentric and isometric muscle work. As suggested in the study by Chen et al. [57], concentric muscle contractions do not cause exercise-induced muscle damage, but exercise-induced muscle damage is evident after eccentric muscle contractions, even in the case of low intensities. Furthermore, the intensity of the proposed balance training was moderate (60%–70% HR max). It was strongly suggested that the increase in Il-6 is related to the duration of exercise and the increase is exponential. It has been shown in research that the level of IL-6 can increase up to 100–fold after strenuous exercise such as a marathon race [17].

Injured muscles recruit monocytes/macrophages via chemotactic factors such as fractalkine. It has been reported that fractalkine induces the reduction of microglia activation and inhibits the release of inflammatory cytokines. In various studies, it has been suggested that fractalkine could play an active role in traumatic and inflammatory neurological diseases, such as traumatic brain injury, MS or ischemic patients. After stroke, in order to limit inflammation, fractalkine release may be a physiological response of the brain tissue to trigger neuroprotection [58]. In the PD study on the rat model, it was revealed that fractalkine reduced the neuronal loss produced by nigrostriatal injection of 6-hydrohydopamine (6-OHDA) [59]. It was proposed that a mechanism underlying fractalkine neuroprotection is its ability to trigger the release of soluble factors that orchestrate a neuroprotective response, among which are adenosine, BDNF and IGF1-1 [60].

Neuroprotective properties have been assigned to anti-inflammatory factor CD200, a transmembrane glycoprotein expressed on neurons which down-regulates inflammatory cytokines and which is involved in the essential signalling pathways in the microglia. Decreased expression of CD200 and its receptor (CD200R) induces inflammation in neurodegenerative disorders [61]. In studies on 6-OHDA-induced rat model of PD, it was revealed that treadmill exercise improved motor balance and increased CD200 expression. In the authors’ study, the aforementioned results were not confirmed. Although the modulatory effect of regular balance exercises of moderate intensity on anti-inflammatory cytokines, BDNF, NGF-β and fractalkine was found in healthy older adults and people with PD, no changes in IGF-1 and CD200 were observed.

The increasing evidence suggests that exercise training, largely due to factors released by contracting muscles, can improve brain functions and plasticity in adults [62]. Exercise may inhibit microglia activation by downregulation of pro-inflammatory cytokines. Exercise-induced downregulation may be interpreted indirectly by upregulation of the level of trophic factors that lead to reduced neuronal injury [5].

A limitation of this study was the relatively small number of participants. HR_max_ was not determined directly during the graded test for each of the subjects, and was estimated from the formula proposed by Tanaka et al. [32]. On this basis, the training range for heart rate corresponding to 60%–70% HR_max_ was determined for each subject individually. Due to the fact that according to the research by [63], HR_peak_ in PD patients may be approximately 5% lower than HR_peak_ in healthy individuals of a similar age, it is most likely that the Parkinson’s disease participants in this study performed about 5% more effort during their training than their healthy peers. However, exercise intensity during training was still moderate in both groups.

## 5. Conclusions

The authors of this study postulate that the attenuation of inflammatory responses after completion of the moderate-intensity balance training could be partly responsible for the observed increase of anti-inflammatory cytokines and neurotrophic factor levels of the participants from training groups. Although with age and disease progression an increase in circulating concentrations of pro-inflammatory cytokines occurs, regular training can modulate the level of inflammatory markers and induce neuroprotective mechanisms to reduce the inflammatory response.

Physical activity may be a natural strategy to improve postural control in healthy older adults and people with PD.

Although we found in the study that regular training can modulate levels of inflammatory markers and induce a neuroprotective mechanism to reduce the inflammatory response, the results are not indicative of improvements in brain function.

The authors assume that the anti-inflammatory and neuroprotective effects of exercise training may be important in modulating disease processes, but mechanisms via which physical activity may be a protective agent against the development of chronic neurological diseases are still waiting to be determined.

## Figures and Tables

**Table 1:** The characteristic of groups.

Variables	PDBT (*n* = 16)	PDNT (*n* = 13)	HBT (*n* = 16)	HNT (*n* = 16)	All PD (*n* = 29)	All H (*n* = 32)	All BT (*n* = 32)	All NT (*n* = 29)
H&Y scale (stage: 2/3)	9/7	9/4			18/11			
Gender (M/F)	11/5	8/5	10/6	10/6	19/10	20/12	21/11	18/11
Age (years)	66.00 ± 2.59	65.23 ± 7.40	67.25 ± 2.52	65.69 ± 3.70	65.66 ± 7.48	66.47 ± 3.21	66.63 ± 7.44	65.48 ± 5.55
Body height (cm)	166.55 ± 7.84	164.88 ± 8.28	165.17 ± 8.99	169.78 ± 9.38	165.80 ± 7.94	167.09 ± 9.24	165.86 ± 8.32	167.16 ± 8.99
Body mass (kg)	81.76 ± 12.29	77.18 ± 14.17	75.09 ± 12.61	80.08 ± 15.01	79.71 ± 13.13	77.58 ± 13.87	78.43 ± 12.71	78.78 ± 14.46
BMI (kg/m^2^)	29.49 ± 4.02	28.20 ± 3.72	27.33 ± 2.16	29.10 (25.29–30.35)	28.91 ± 3.87	27.45 (26.10–29.30)	28.41 ± 3.36	29.00 (25.90–30.60)

Data presented as mean ± SD or median (IQR); significant differences *p* < 0.05 (PDBT vs. PDNT vs. HBT vs. HNT: Test F analysis of variance or Kruskal–Wallis Test; All PD vs. All H or All BT vs. All NT: Student’s test or Mann–Whitney U test); SD: standard deviation; IQR: interquartile range; PD: Parkinson Disease; H: healthy; BMI: Body mass index; BT: balance training; H&Y: Hoehn and Yahr Scale; M: male; F: female, HBT: group of training, healthy older individuals; HNT: group of nontraining older, healthy individuals; PDBT: training group with PD; PDNT: nontraining PD group.

**Table 2:** Influence of balance training with moderate-intensity exercises in persons with Parkinson’s disease and healthy older individuals on the postural control outcome compared with control groups.

									Baseline Characteristic	
Variables	Training	PDBT (*n* = 16)	PDNT (*n* = 13)	HBT (*n* = 16)	HNT (*n* = 16)	GROUP	F (*p*) BT	GROUP × BT	All PD (*n* = 29)	All Healthy (*n* = 32)
POMA (points)	pre BT	21.38 ± 2.25 ‡^§^	21.31 ± 2.59 ^#^†	25.94 ± 1.57	27.31 ± 1.14	58.05	29.05	12.72	21.34 ± 2.36 ^a^	26.63 ± 1.52
	after BT	23.13 ± 2.16 *‡^§&^	20.62 ± 2.66 ^#^†	28.00 ± 0.00 *	27.88 ± 0.62	(<0.01)	(<0.01)	(<0.01)		

Data presented as mean ± SD; * significant influence of BT (*p* < 0.05); ^§^: PDBT group vs. HBT group (*p* < 0.05); ‡: PDBT group vs. HNT group (*p* < 0.05); ^#^: PDNT group vs. HNT group (*p* < 0.05); †: PDNT group vs. HBT group (*p* < 0.05); ^&^ PDNT group vs. PDBT group (*p* < 0.05); ^a^: All PD group vs. All H group (*p* < 0.05); SD: standard deviation; PD: Parkinson Disease; H: healthy; BT: balance training; HBT: group of training, healthy elder individuals; HNT: group of non-training elder, healthy individuals; PDBT: training group with PD; PDNT: non-training PD group.

**Table 3:** Influence of balance training with moderate-intensity exercises in persons with Parkinson’s disease and healthy older individuals on the concentration of selected cytokines, neurotrophic factors and CD200 proteins as well as fractalkine compared with control groups.

									Baseline Characteristic	
Variables	Training	PDBT (*n* = 16)	PDNT (*n* = 13)	HBT (*n* = 16)	HNT (*n* = 16)	GROUP	F (*p*) BT	GROUP × BT	All PD (*n* = 29)	All Healthy (*n* = 32)
IL-6 (pg/mL)	pre BT	2.08 ± 1.31	2.13 ± 0.81	2.51 ± 1.20	2.56 ± 1.27	1.36	0.84	0.55	2.10 ± 1.10	2.54 ± 1.21
	after BT	2.27 ± 1.14	2.21 ± 1.51	2.39 ± 0.95	3.06 ± 1.44	(0.26)	(0.36)	(0.64)		
IL-10 (pg/mL)	pre BT	2.75 ± 1.09	2.59 ± 1.52	2.82 ± 1.67	2.71 ± 1.15	1.08	10.05	2.41	2.68 ± 1.28	2.77 ± 1.41
	after BT	3.76 ± 2.29 *	2.69 ± 1.36 †	4.42 ± 3.42 *	2.92 ± 0.95 ^$^	(0.36)	(<0.01)	(0.07)		
TNF-α (pg/mL)	pre BT	1.06 ± 0.28	0.90 ± 0.33	0.97 ± 0.19	1.23 ± 0.39	4.09	0.17	2.84	0.99 ± 0.31	1.10 ± 0.33
	after BT	0.89 ± 0.30 *‡	0.92 ± 0.19 ^#^	1.06 ± 0.29 ^$^	1.27 ± 0.45	(0.01)	(0.89)	(0.04)		
BDNF (ng/mL)	pre BT	21.19 ± 8.36	30.08 ± 8.04	20.21 ± 13.33	28.10 ± 12.33	0.51	11.52	4.56	25.17 ± 9.24	24.28 ± 13.23
	after BT	30.37 ± 6.33 *	25.78 ± 11.72	34.98 ± 20.62 *	33.13 ± 17.74	(0.67)	(<0.01)	(0.01)		
β-NGF (pg/mL)	pre BT	109.42 ± 87.14 ‡^§^	114.73 ± 66.06 †^#^	310.42 ± 156.88	345.00 ± 162.32	12.92	10.14	1.58	111.80 ± 77.11 ^a^	327.71 ± 158.01
	after BT	224.83 ± 194.20 *‡ ^§^	131.36 ± 104.00 †^#^	459.36 ± 212.27 *	385.05 ± 219.94	(<0.01)	(<0.01)	(0.20)		
TGF-β1 (ng/mL)	pre BT	21.54 ± 5.27	27.04 ± 7.10	19.21 ± 8.22	23.46 ± 14.17	0.14	3.36	4.65	24.01 ± 6.65	21.34 ± 11.59
	after BT	27.88 ± 11.02 *	21.45 ± 7.60	26.97 ± 7.25 *	25.22 ± 7.30	(0.93)	(0.06)	(<0.01)		
IGF-I (ng/mL)	pre BT	180.62 ± 51.60	183.20 ± 65.44	143.68 ± 37.78	163.78 ± 46.03	1.80	1.86	2.00	181.78 ± 57.13 ^a^	153.73 ± 42.67
	after BT	211.36 ± 117.61	180.04 ± 69.46	165.21 ± 40.90	151.94 ± 37.98	(0.15)	(0.17)	(0.12)		
CD200 (pg/mL)	pre BT	102.86 ± 77.20	128.89 ± 84.31	91.39 ± 78.69	89.43 ± 69.63	0.59	0.00	0.45	114.53 ± 80.08	90.41 ± 73.01
	after BT	101.75 ± 44.22	115.25 ± 84.21	105 ± 73 ± 97.18	87.69 ± 67.58	(0.62)	(0.99)	(0.72)		
Fractalkine (ng/mL)	pre BT	0.44 ± 0.06	0.47 ± 0.11	0.42 ± 0.11	0.42 ± 0.11	0.50	5.20	3.04	0.46 ± 0.09	0.42 ± 0.11
	after BT	0.48 ± 0.07 *	0.45 ± 0.13	0.48 ± 0.15 *	0.43 ± 0.08	(0.68)	(0.02)	(0.03)		

Data presented as mean ± SD; * significant influence of BT (*p* < 0.05); ^§^: PDBT group vs. HBT group (*p* < 0.05); ‡: PDBT group vs. HNT group (*p* < 0.05); ^#^: PDNT group vs. HNT group (*p* < 0.05); †: PDNT group vs. HBT group (*p* < 0.05); ^$^: HBT group vs. HNT group (*p* < 0.05); ^a^: All PD group vs. All H group (*p* < 0.05); SD: standard deviation; PD: Parkinson Disease; H: healthy; BT: balance training; HBT: group of training, healthy older individuals; HNT: group of nontraining older, healthy individuals; PDBT: training group with PD; PDNT: nontraining PD group; IL-6: interleukin 6; IL-10: interleukin 10; TNF-α: tumor necrosis factor alpha; BDNF: brain-derived neurotrophic factor; β-NGF: beta nerve growth factor; TGF-β1: beta 1 transforming growth factor;IGF-1: insulin-like growth factor 1.
